# Supplementary material for: Genetic determinants of COVID-19 severity and mortality: ACE1 Alu 287 bp polymorphism and ACE1, ACE2, TMPRSS2 expression in hospitalized patients
Source: PeerJ. 2025 Jan 20;13:e18508. doi: 10.7717/peerj.18508 (PMC11756369; doi:10.7717/peerj.18508)
Supplement: Supplemental Information 2 [file peerj-13-18508-s002.doc]

STROBE Statement—Checklist of items that should be included in reports of ***case-control studies***

|  | Item No | Recommendation | Position in the text |
| --- | --- | --- | --- |
| **Title and abstract** | 1 | (*a*) Indicate the study’s design with a commonly used term in the title or the abstract | Line 1 |
|  |  | (*b*) Provide in the abstract an informative and balanced summary of what was done and what was found | Lines 32-53 |
| Introduction | |  |  |
| Background/rationale | 2 | Explain the scientific background and rationale for the investigation being reported | Lines 81-85 |
| Objectives | 3 | State specific objectives, including any prespecified hypotheses | Lines 83-85 |
| Methods | |  |  |
| Study design | 4 | Present key elements of study design early in the paper | Lines 87-94 |
| Setting | 5 | Describe the setting, locations, and relevant dates, including periods of recruitment, exposure, follow-up, and data collection | Lines 87-100 |
| Participants | 6 | (*a*) Give the eligibility criteria, and the sources and methods of case ascertainment and control selection. Give the rationale for the choice of cases and controls | Lines 101 - 106 |
|  |  | (*b*) For matched studies, give matching criteria and the number of controls per case | Not applicable. |
| Variables | 7 | Clearly define all outcomes, exposures, predictors, potential confounders, and effect modifiers. Give diagnostic criteria, if applicable | Lines 87 - 106 |
| Data sources/ measurement | 8* | For each variable of interest, give sources of data and details of methods of assessment (measurement). Describe comparability of assessment methods if there is more than one group | Lines 96 - 97 |
| Bias | 9 | Describe any efforts to address potential sources of bias | Lines 102 -107 |
| Study size | 10 | Explain how the study size was arrived at | Lines 87 - 91 |
| Quantitative variables | 11 | Explain how quantitative variables were handled in the analyses. If applicable, describe which groupings were chosen and why | Lines 130 - 137 |
| Statistical methods | 12 | (*a*) Describe all statistical methods, including those used to control for confounding | Lines 130 - 137 |
|  |  | (*b*) Describe any methods used to examine subgroups and interactions | Not applicable. |
|  |  | (*c*) Explain how missing data were addressed | Lines 130 - 137 |
|  |  | (*d*) If applicable, explain how matching of cases and controls was addressed | Lines 130 – 137 |
|  |  | (*e*) Describe any sensitivity analyses | Not applicable. |
| Results | |  |  |
| Participants | 13* | (a) Report numbers of individuals at each stage of study—eg numbers potentially eligible, examined for eligibility, confirmed eligible, included in the study, completing follow-up, and analysed | Tabela 1 |
|  |  | (b) Give reasons for non-participation at each stage | Not applicable. |
|  |  | (c) Consider use of a flow diagram | Not applicable. |
| Descriptive data | 14* | (a) Give characteristics of study participants (eg demographic, clinical, social) and information on exposures and potential confounders | Tabela 1 |
|  |  | (b) Indicate number of participants with missing data for each variable of interest | Tabela 1 |
| Outcome data | 15* | Report numbers in each exposure category, or summary measures of exposure | Table 1, 2, 3, and 4 |
| Main results | 16 | (*a*) Give unadjusted estimates and, if applicable, confounder-adjusted estimates and their precision (eg, 95% confidence interval). Make clear which confounders were adjusted for and why they were included | Lines 151 - 177 |
|  |  | (*b*) Report category boundaries when continuous variables were categorized | Not applicable. |
|  |  | (*c*) If relevant, consider translating estimates of relative risk into absolute risk for a meaningful time period | Not applicable. |

| Other analyses | 17 | Report other analyses done—eg analyses of subgroups and interactions, and sensitivity analyses | Not applicable. |
| --- | --- | --- | --- |
| Discussion |  |  |  |
| Key results | 18 | Summarise key results with reference to study objectives | Lines 192 - 195 |
| Limitations | 19 | Discuss limitations of the study, taking into account sources of potential bias or imprecision. Discuss both direction and magnitude of any potential bias | Lines 204 - 210 |
| Interpretation | 20 | Give a cautious overall interpretation of results considering objectives, limitations, multiplicity of analyses, results from similar studies, and other relevant evidence | Lines 212 - 218 |
| Generalisability | 21 | Discuss the generalisability (external validity) of the study results | Lines 216 - 218 |
| Other information |  |  |  |
| Funding | 22 | Give the source of funding and the role of the funders for the present study and, if applicable, for the original study on which the present article is based | Lines 237 - 242 |

*Give information separately for cases and controls.

**Note:** An Explanation and Elaboration article discusses each checklist item and gives methodological background and published examples of transparent reporting. The STROBE checklist is best used in conjunction with this article (freely available on the Web sites of PLoS Medicine at http://www.plosmedicine.org/, Annals of Internal Medicine at http://www.annals.org/, and Epidemiology at http://www.epidem.com/). Information on the STROBE Initiative is available at http://www.strobe-statement.org.
