# Supplementary figures and images for: Genetic determinants of COVID-19 severity and mortality: ACE1 Alu 287 bp polymorphism and ACE1, ACE2, TMPRSS2 expression in hospitalized patients
Source: PeerJ. 2025 Jan 20;13:e18508. doi: 10.7717/peerj.18508 (PMC11756369; doi:10.7717/peerj.18508)

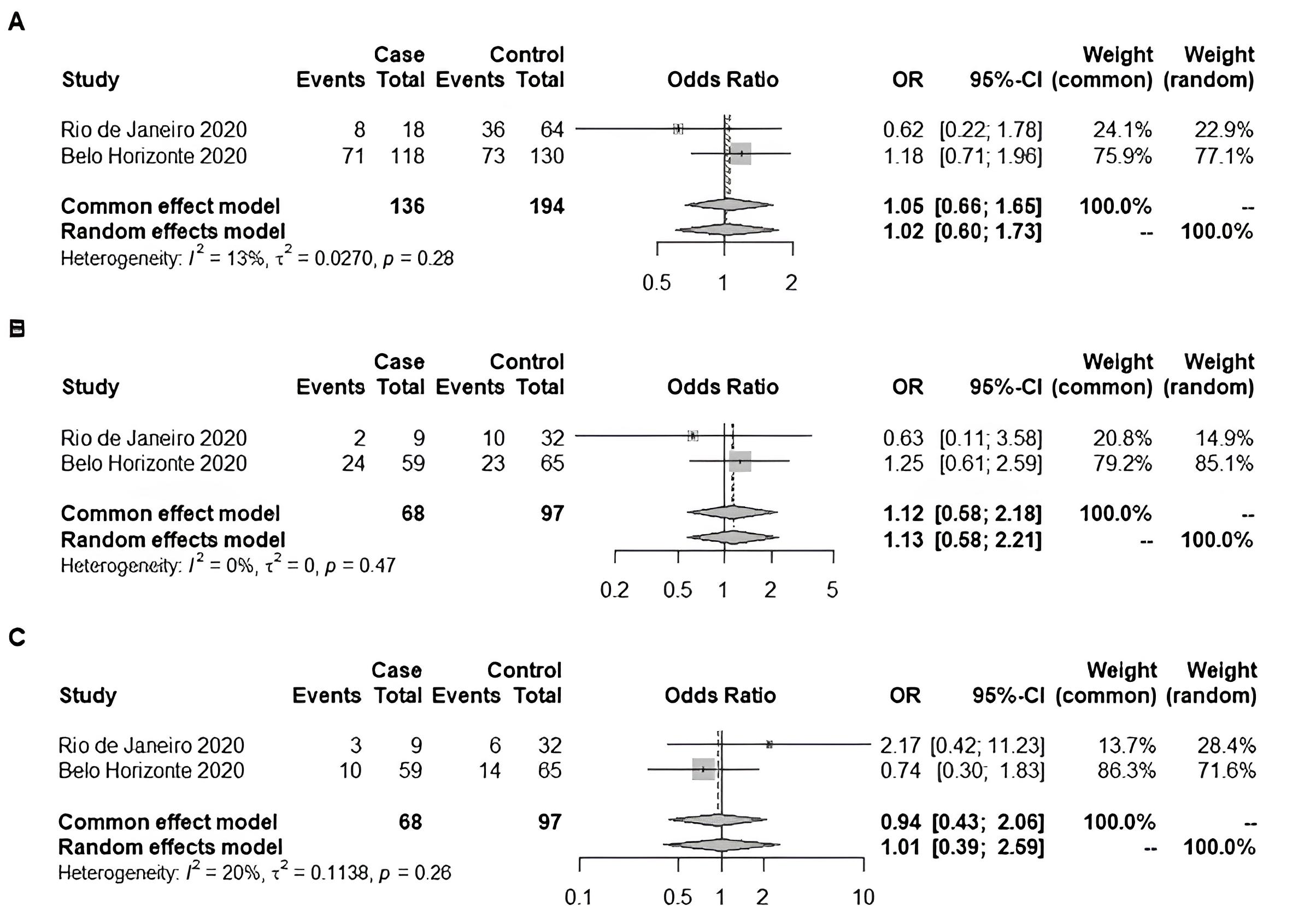

Supplement: Figure S1 — The effect sizes from the two cohorts (Rio de Janeiro and Belo Horizonte) were combined. No significant allelic or genotypic effects were observed under the random-effects model. Case and control definitions are presented in Table 3. (A) D-allele model: Effect of the D-allele on the need for mechanical ventilation. (B) D recessive model: Effect of the D/D genotype on the need for mechanical ventilation compared to the combined D/I and I/I genotypes. (C) I recessive model: Effect of the I/I genotype on the need for mechanical ventilation compared to the combined D/I and D/D genotypes. Statistical significance was assessed using the Mantel–Haenszel weighted means method under the fixed-effect model. [file peerj-13-18508-s003.jpg]

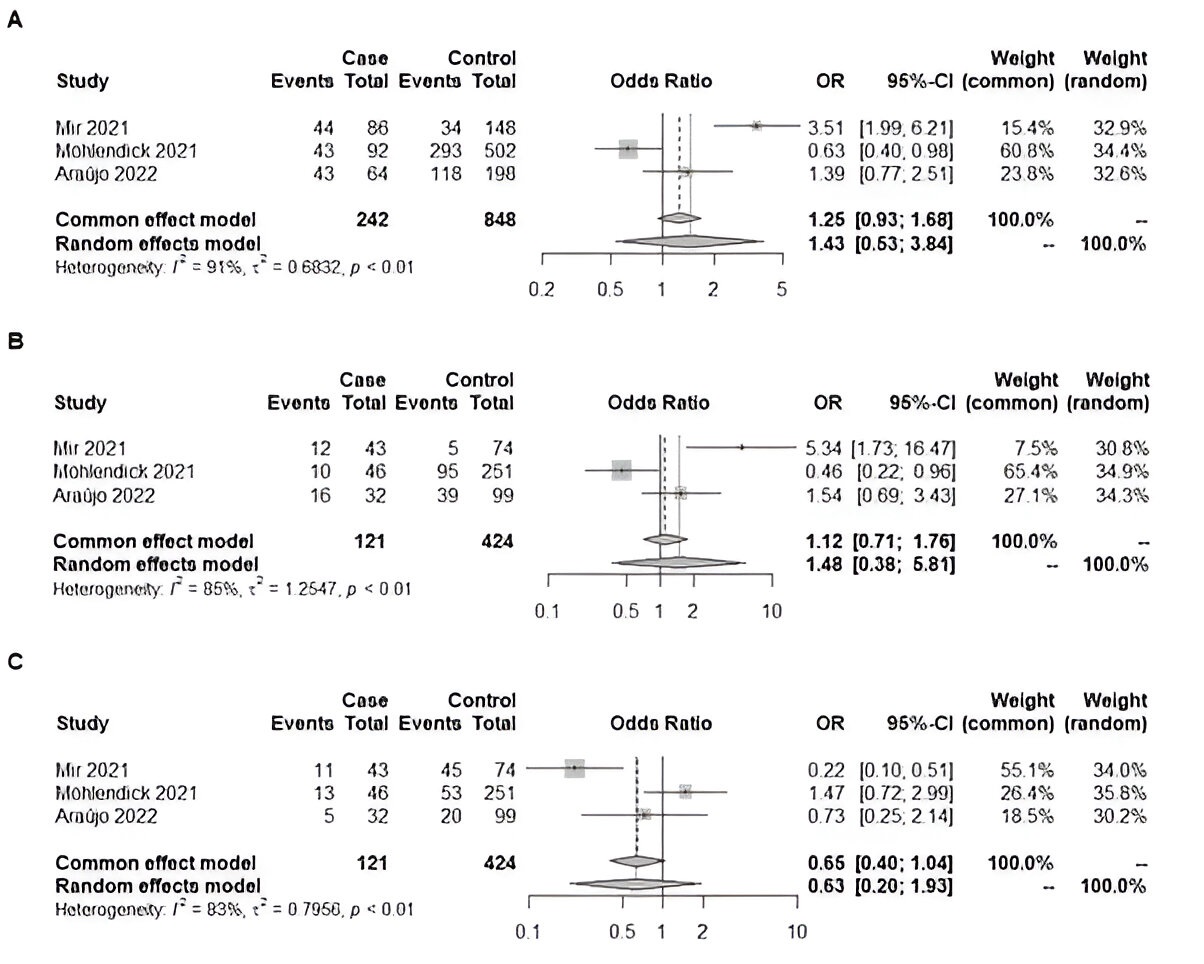

Supplement: Figure S2 — The effect size from our original study was combined with two additional studies from the literature. No significant allelic or genotypic effects were observed under the random-effects model. Case and control definitions are presented in Table 2. (A) D-allele model: Effect of the D-allele on the risk of death. (B) D recessive model: Effect of the D/D genotype on the risk of death compared to the combined D/I and I/I genotypes. (C) I recessive model: Effect of the I/I genotype on the risk of death compared to the combined D/I and D/D genotypes. Statistical significance was assessed using the Mantel–Haenszel weighted means method under the fixed-effect model. [file peerj-13-18508-s004.jpg]
